# Comparative Evaluation of Speech Quality Before and After Functional Palatal Recontouring in Complete Dentures using Artificial Intelligence: A Study Protocol

**DOI:** 10.7759/cureus.69127

**Published:** 2024-09-10

**Authors:** Madhu Priya, Surekha A Dubey, Jahnavi P Gorripati

**Affiliations:** 1 Prosthodontics, Sharad Pawar Dental College and Hospital, Datta Meghe Institute of Higher Education and Research, Wardha, IND

**Keywords:** artificial intelligence in dentistry, palatogram, palatograph, phonetics, speech quality

## Abstract

Introduction

In the realm of dentistry, where precision and understanding of oral structures are paramount, the utilization of advanced technologies has revolutionized diagnostic and treatment methodologies. One such technology, palatography, holds significant importance in understanding the intricate dynamics of the oral cavity, particularly the interaction between the tongue and the palate during speech and swallowing. In the field of prosthodontics, achieving a successful outcome goes beyond simply restoring missing teeth. A well-designed denture or prosthesis should not only function properly but also consider factors like aesthetics, comfort, and speech intelligibility.

Methods

The speech quality analysis using artificial intelligence has been designed to assist in the palatal recontouring of complete dentures during the trial denture appointment and denture insertion. The study will be conducted on 12 completely edentulous patients, selected based on exclusion and inclusion criteria.

Expected results

The palatal recontouring assessment is expected to improve speech quality significantly. This could be incorporated into routine complete denture rehabilitation cases where the ease of technology can help enhance the phonetics of the prosthesis.

Conclusions

Patients undergoing full denture rehabilitation benefit greatly from phonetics. Taking this key factor into play through the use of advanced but readily available technology, palatography can be employed as a routine procedure for rehabilitation through complete dentures. There is definitely a learning curve involved while making it a routine procedure, but it is, nonetheless, a significant addition to holistic rehabilitation.

## Introduction

Speech is essential to human activity because it is a vital part of the stomatognathic system, which uses the oral cavity as a component of the voice. The vocal cords vibrate with the expiratory airflow allowing the larynx to create voice. Vowel formants are conditioned by the geometry of the resonance cavities, which in turn modulate the vocal cord tension, determining the laryngeal tone frequency. The tongue, gums, palate, alveolar processes, lips, and teeth are the speech articulatory organs [1,2]. Phonetics has to be seen alongside mechanics and aesthetics as the primary elements influencing dental prosthesis success [2,3].

It is important to make dentures that allow the patient to speak and voice themselves without any difficulties. Changes in the vertical dimension, size, and placement of the teeth, as well as the thickness and shape of the denture base, can all contribute to articulatory mistakes [1,2]. If other aspects of the maxillary full denture such as tooth location, occlusal vertical dimension, and occlusal plane are adequate, then accurately approximating the palatal contours with the patient's tongue can enhance speech intelligibility [4]. Maxillary dentures' artificial palatal vaults frequently have a concave contour. On the other hand, naturally occurring palatal vaults in the alveolar area have a convex form. Therefore, within a week following the placement of new dentures, the palatal vaults of maxillary dentures need to resemble natural ones in form to promote accurate pronunciation [5]. 

Research shows that many patients place a high value on speech sound production being undisturbed following dental treatment. The way that patients produce speech affects how satisfied they are with their dentures overall [6]. The aim of this study is to improve phonetics in a complete denture by functional palatal contouring during the try-in stage to improve the quality of life of patients.

## Materials and methods

Proposed study design

This experimental study will be conducted in the Department of Prosthodontics and Crown & Bridge, Sharad Pawar Dental College and Hospital, Wardha, Maharastra, India. The duration of the study will be six months. A total of 12 patients would be included in the study based on the inclusion and exclusion criteria (Table 1).

**Table 1:** Inclusion and exclusion criteria

Inclusion Criteria	Exclusion Criteria
Patients indicated for a complete denture prosthesis.	Patient with macroglossia
Patients with Angles’s type 1 ridge relation.	Patients with congenital speech difficulty

Intended procedure

**Figure 1:**
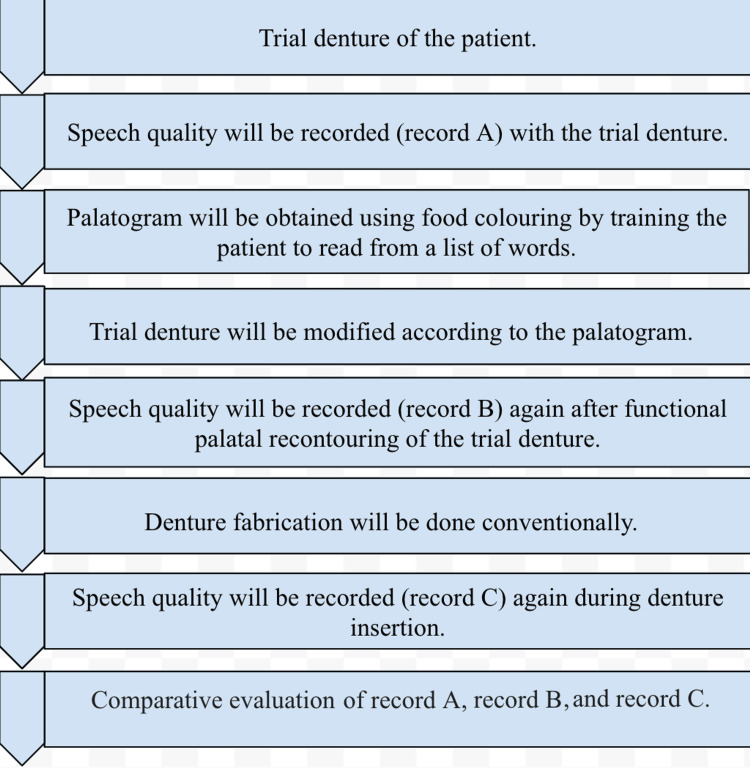
Flowchart of the study

On the day of the trial denture appointment for the complete denture rehabilitation patient, the patient will be asked to read from a list of words. This will be recorded (Record A) through a microphone connected to artificial intelligence (AI) software, which will analyze the speech quality. After this, for palatography, food coloring will be applied to the palatal surface of the trial denture, and the patient will be asked to read from the previous list of words [7,8]. Based on the palatogram obtained using the food coloring, the palatal re-contouring would be done on the trial denture. Speech quality will be recorded again in the previously stated manner (Record B). After this, the denture fabrication will be done in a conventional manner. On the denture insertion appointment day, another recording (Record C) would be done wearing the complete denture in the same manner as previously stated. The values obtained from the AI software will be compared and analyzed statistically (Figure 1).

Sample size

Mean difference formula was used to determine the sample size, n=[{(Z𝛼 + Z𝛽)2 𝛔2}/𝛿2], where Z𝛼 = 1.96 at 5% error and 95% confidence interval and Z𝛽 = 0.84 = Power at 99%. The primary variable is selected as Sh (Hz) linguopalatal contact and Wide Air Blade. Mean in Group 1 = 1170.6 ± 65.1. Mean in Group 2 = 1291.0 ± 67.8. Mean score difference (1291.0 - 1170.6) = 120.4. Pooled standard deviation = 66.45. Minimum sample size, n, = 12/record (group). As there will be three recordings, the total samples will be 12x3= 36.

Statistical analysis

An ANOVA test will be used to find the significant difference between the three groups for their mean evaluation of speech quality at the 5% level of significance.

Ethical considerations

The Datta Meghe Institute of Higher Education and Research's Institutional Ethics Committee has approved this study (approval number: DMIHER(DU)/IEC/2024/55) dated May 11, 2024. Research ethics rules shall be followed at all times, and participant anonymity will be protected.

Trial registration

The study was registered with the Clinical Trial Registry-India (CTRI) on July 9, 2024 (registration number: CTRI/2024/07/070341).

## Results

This study will elicit the advantages of functional palatal re-contouring and the use of an easily accessible and readily available technology. It is aimed at improving the phonetics of complete denture patients, as most of the palato-lingual sounds are compromised due to the palatal coverage of the dentures. The scope of this study is to establish functional palatal contouring as a protocol during the fabrication of a complete denture prosthesis for more holistic rehabilitation. This is an ongoing study protocol, and the results are going to be collected and analyzed in early 2025.

## Discussion

Palatal re-contoring has been done using various methods, but due to the complexity of the devices used to analyze speech quality, it is seldom used in everyday practice. Using auditory analyzer software, Bizyaev et al. examined the phonetics of patients with permanent dentures [9]. Prior to receiving orthopedic therapy, patient sonograms and spectrum analyses were used as research data. The type of mispronounced speech sound fluctuated based on the palatal vault and the angulation of the upper front teeth caused by the palatal laminates. The results of instrumental acoustic analysis, which were obtained using specialized auditory analyzer software, attest to the notable progress in the pronunciation of speech sounds whose origins are in the anterior region of the upper front teeth. Orthopedists and dentists can benefit from these instrumental acoustic analyses and spectrogram sonograms that use computer technology [9].

Abu-Awwad et al., in their study, included 10 healthy edentulous individuals aged 45-80 years and convention dentures were used. Speech recordings were made in order to analyze the speech's acoustics and for intelligibility. The following denture options were recorded: no denture, standard denture, random rugae denture, and customized rugae denture. A 60-day gap separated each recording session. A paired "t" test was used for the analysis and comparison of all four recordings. In rugae-integrated dentures, there was a noticeable improvement in the frequency peak noise energy for the letter "s" and the anti-formant frequency for the letter "n." With rugae integrated dentures, there was a relative improvement in voice onset time for "d," frequency proximity burst, and frequency peak noise energy for "sh." Substitution mistakes with traditional dentures have been observed in the findings of the intelligibility study. With rugae-integrated dentures, the speech was somewhat improved. Among these, rugae dentures that were specially made outperformed rugae dentures made at random [10].

Using a computerized speech lab (spectrogram), the study by Mahross et al. examined the effects of reproducing varying thickness and palatal rugae materials on speech produced by full dentures [11]. Three male patients, who were entirely edentulous and between the ages of 50 and 60, were chosen to read a paragraph. Four maxillary dentures were used for each patient, a total of 12. Five speech groups were created with these three patients with the different dentures: Group I was without dentures; Group II was with conventional acrylic dentures for rehabilitation; Group III included conventional dentures with reproduction of rugae; Group IV was with dentures that had a minimally thick metallic framework and rough surface at rugae; and Group V used acrylic material, thinner than the denture, for their rugae. Following the placement of each denture for each group, speech samples were captured using the spectrogram. The lingo-palatal sounds /s/z/sh/t/d/ and /l/ were chosen. Using the /sh/t/ sound, Group III generated a high mean meaningful difference. Group V showed a difference with the z/l sound (p < 0.05), whereas Group IV showed a difference with the linguo-palatal sounds. The conclusion reached was that since the entire denture with rugae has a better phonetic quality than a conventional denture, it is advised to replicate the rugae region [11]. 

Chaturvedi et al. conducted an in vivo study to assess the quality of speech sounds (i.e., pitch and intensity) in four stages: phase I-an edentulous state; phase II-a traditional full denture; phase III-a functionally contoured palatal base denture; and phase IV-dentures with a little aperture in the anterior region of the palatal base [12]. There were 40 edentulous patients in all. Type I Modification: Functionally Contoured Modified Palate, or FCMP and Type II Small-opening (SO) modification at the palatal base's anterior portion. It was determined that following denture placement, speech intelligibility was more prevalent and improved in dentures that had been changed (Type I and II modifications). Speech sounds generated by dentures with Modification I showed a relative improvement in clarity, whereas full dentures with Modification II showed a considerable improvement. The speech sounds produced were highly clear and did not require any adaptation time following denture implantation [12].

Bulycheva et al.'s study evaluated the pronunciation of individuals with complete edentulous arches, both after and before prosthodontics therapy utilizing full removable dentures. Eighty-one patients, aged 35-79, were evaluated (39 males and 42 females) [13]. The prosthetic treatment group formed the experimental group, while patients who had normal occlusion and undamaged teeth were the control group. One month following their prosthetic therapy, the patients in the experimental group had their recordings produced. They experienced speech impairments such as lisping, noises, and whistling sounds. Sound properties can be objectively, subjectively, and quantitatively analyzed using spectrum analysis of sound pronunciation. A useful technique for the differential identification of problems related to sound production is the spectral analysis of sounds recorded both before and after prosthodontics therapy. In order to evaluate prominent sounds objectively, qualitatively, and quantitatively, as well as the effectiveness of therapy, spectral analysis may need to be used for disorders of phonation. 

Study limitation

The study will be conducted to establish speech quality assessment using AI, and will be a technology-based and limited application study with a limited sample size. A future study should be conducted with a larger sample size for further investigation.

## Conclusions

Phonetics plays an important role in the complete denture rehabilitation of patients. Taking this key factor into play through the use of advanced but readily available technology, palatography can be employed as a routine procedure for the fabrication of complete dentures. There is definitely a learning curve involved in making it a routine procedure, but nonetheless, it is a significant addition to holistic rehabilitation.
